# Emergency resuscitative thoracotomy performed in European civilian trauma patients with blunt or penetrating injuries: a systematic review

**DOI:** 10.1007/s00068-015-0559-z

**Published:** 2015-08-18

**Authors:** J. K. Narvestad, M. Meskinfamfard, K. Søreide

**Affiliations:** 1 Department of Gastrointestinal Surgery, Stavanger University Hospital, POB 8100, 4068 Stavanger, Norway; 2 Department of Clinical Medicine, University of Bergen, Bergen, Norway

**Keywords:** Emergency thoracotomy, Resuscitation, Survival, Blunt trauma, Penetrating trauma

## Abstract

**Purpose:**

Emergency resuscitative thoracotomy (ERT) is a lifesaving procedure in selected patients. Indications are still being debated, but outcome in blunt trauma is believed to be poor. Recent reports from European populations, where blunt trauma predominates, have suggested favorable outcome also in blunt trauma. Our aim was to identify all European studies reported over the last decade and compare reported outcomes to existing knowledge.

**Methods:**

We performed a systematic literature search according to PRISMA guidelines (January 1st, 2004 to December 31st, 2014). The “grey literature” was included by searching Google Scholar. Qualitative comparison of studies and outcomes was done.

**Results:**

A total of 8 articles from Europe were included originating from Croatia, Norway (*n* = 2), Denmark, Iceland, the Netherlands, Scotland, and Switzerland. Of 376 resuscitative thoracotomies, 193 (51.3 %) were for blunt trauma. Male:female distribution was 3.5:1. The collectively reported overall survival was 42.8 % (*n* = 161), with 25.4 % (49 of 193) blunt trauma and 61.2 % (112 of 183) penetrating injuries. When strictly including those ERTs designated as done in the emergency department for blunt mechanism (*n* = 139) only, a total of 18 patients survived (12.9 %). Survival after EDTs for penetrating trauma was 41.6 % (37 of 89). Neurological outcome (reported in 5 of 8 studies) reported favorable neurological long-term outcome in the majority of survivors, even after blunt trauma. None referred to Glasgow Outcome Score. Heterogeneity in the studies prevented outcome analyses by formal quantitative meta-analysis.

**Conclusion:**

The reported outcome after ERT in European civilian trauma populations is favorable, with one in every four ERTs in the ED surviving. Notably, outcome is at variance with previously reported collective data, in particular for blunt trauma. Multicenter, prospective, observational data are needed to validate the modern role of ERT in blunt trauma.

## Introduction

Emergency resuscitative thoracotomy (ERT) may serve as a lifesaving procedure for selected trauma patients presenting in extremis with pending or already witnessed cardiopulmonary collapse. Since the 1960s, the pendulum for resuscitative emergency thoracotomy has swung from conservative to a more aggressive approach, but the use, indications and risks are still debated [1–4]. Collective evidence and consensus have suggested that outcome is best for penetrating trauma patients with pending (or witnessed) cardiac arrest, with most favorable outcomes reported for patients with an isolated stab wound to the heart [2, 5–7]. Notably, the majority of studies published stem from large-volume institutions in North America or South Africa, where penetrating trauma represents a predominating injury mechanism and for which surgeons and systems are readily trained to deal with these injuries [2, 8]. Contrary to the experience in regions with a high incidence of penetrating trauma, the predominant mechanism seen in the majority of European hospitals is related to blunt injuries, with even busy centers receiving far less than 10 % penetrating trauma. Blunt trauma victims are generally believed to have a poor outcome if cardiac arrest follows or circulatory collapse is pending. An ERT is believed to be rarely indicated in blunt trauma with cardiac arrest as outcomes are dismal in the vast majority [2].

However, with the maturation of systems across Europe, an increasing reported series of resuscitative emergency thoracotomies from European populations have accumulated, even with successful experience from pre-hospital employment in one series [9] and accumulating experience also from the recent wars [10]. However, collectively, little is known about the outcome of resuscitative ET in the modern civilian European trauma populations besides anecdotal reports. Thus, we wanted to systematically review the reported experience and outcomes of ERT use in these civilian trauma populations and compare the results from the reported literature.

The aim of the study was to give a systematic overview of reported indications, outcomes and reports based on civilian European publications over the past decade. Secondly, we wanted to evaluate the standardized reported variables, factors and outcomes related to these reports.

## Materials and methods

### Search methods and inclusions and exclusion criteria

We performed a systematic literature search according to the PRISMA guidelines [11] of the worldwide literature in PubMed/MEDLINE and EMBASE databases using the keywords and/or medical Subject headings (MeSH) terms “emergency thoracotomy”, “emergency department thoracotomy”, “resuscitative thoracotomy”, “urgent thoracotomy”, “Trauma”, “Europe”. The study was limited to the time period of January 2004 to December 2014 to present updated experiences and avoid dated reports from the past. Additional searches of other databases (EMBASE, etc.) were performed by a trained hospital librarian. The international database of prospectively registered systematic reviews (PROSPERO) was queried for potential planned or ongoing systematic reviews.

Titles and abstracts of studies were scrutinized for relevance. We included any report published in the English, German, or Scandinavian (Norwegian, Danish, Swedish) languages, and considered studies in French, Spanish or other European languages if sufficient information was obtained in English abstract form or, if further detailed information could be retrieved by contacting the authors.

The references of the identified fulltext articles were further hand-searched to retrieve additional studies. The ‘grey literature’ was searched using Google and Google Scholar to identify studies published outside the PubMed index, including the *European Journal of Trauma and Emergency Surgery*.

We excluded publications on the following criteria: case reports on only one or, only a couple of cases; reports of few cases (<5 ERTs); reports from military medicine (e.g. the war in Afghanistan and Iraq); and, reports on pre-hospital resuscitative thoracotomy.

Based on small population samples, the high likelihood for heterogeneity and risk of bias in the included studies, we deferred formal meta-analysis and, rather, focused on the descriptive data and outcomes retrieved in the included reports.

### Definitions used and data collected for each study

There is no standard definition to Emergency Resuscitative Thoracotomy (ERT) in the literature and investigators use interchangeably the terms ‘resuscitative thoracotomy’, ‘emergency thoracotomy’, ‘emergency department thoracotomy’, ‘urgent thoracotomy’ and other combinations thereof, either focusing on the location of the procedure or the urgency of its indication. We scrutinized the definitions used in each paper as located either in emergency department or operating room, and as emergent or urgent in nature, and included papers that clearly reported on the procedure as part of the resuscitative or emergency management process of the trauma patient, either located in the emergency department (ED) or the operating room (OR). For consistent reading, we chose the term ‘ERT’ throughout the paper.

Demographic data such as geography, number of patients, age and gender were recorded for included studies.

Mechanism of injury (MOI) was defined as either blunt or penetrating. Location of major injury (LMOI) was limited to the major anatomic area of injury as cerebral, abdominal, thoracic, pelvic and/or multiple.

Injury severity was obtained from each study as reported by the injury severity score (ISS) [12] and probability of survival (Ps) as per calculation by TRISS methodology [13], if given.

*Survival* was obtained for each study for the entire cohort or, alternatively, for the subcohorts, as reported.

The following measures were further searched for in each paper:

- Indications for ERT, whether reported and, if yes, what type of indication reported (e.g. cardiac tamponade, shock, intraabdominal hemorrhage, etc.).
- Signs of life (SOL) if documented and how defined in each study.
- Vital signs, if documented and how defined.
- Neurological outcome, whether investigated and reported and, if yes, by what means reported (either as “good/poor”) or by any formal score, such as Glasgow Outcome Score (GOS) [14].

### Data presentation and statistical analyses

Data are presented in a descriptive manner and as reported in the respective studies. We assumed upfront that studies would be of a very heterogeneous nature and thus did not plan to perform a formal meta-analyses, as this would not be substantiated based on the very high risk of bias.

## Results

**Fig. 1:**
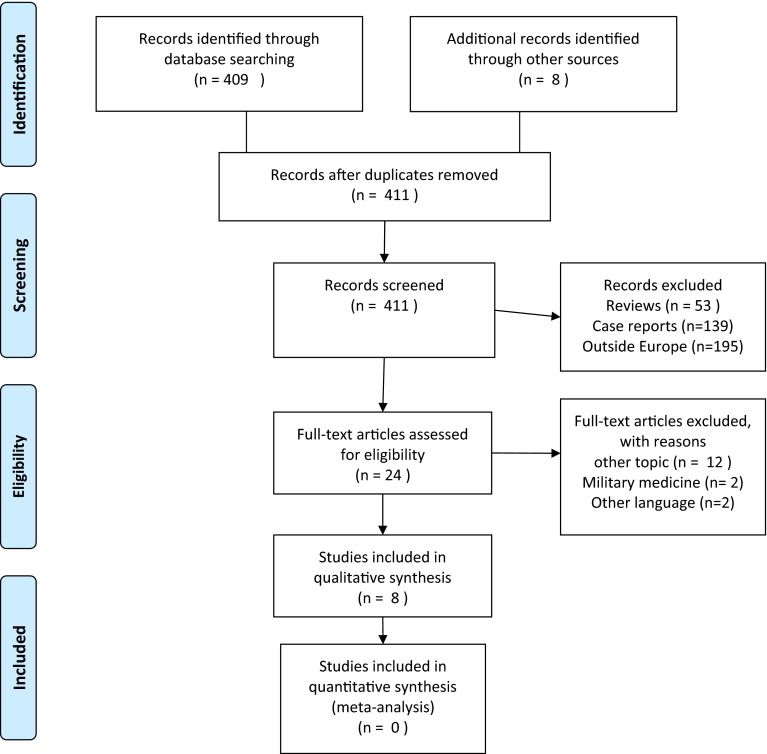
PRISMA flowchart of included studies

The search result is shown in Fig. 1. For the study period, we found a total of 8 studies [15–22] that originated from Europe and for which the defined inclusion criteria were fulfilled. The study descriptives, patient demographics and main findings are reported in Table 1. A further two publications were found from Norway and Ireland [23, 24]. Upon author contact, we were informed that 5 resuscitative emergency thoracotomies were performed, with no survivors in Tromsø, Norway. Contact with the group from Dublin failed. Due to a lack of detailed information from these sites, we could not include these studies in the collective presentation. A register study from Germany reported outcome on a subset of patients having emergency thoracotomy, but with no specific details to the group as such, and was thus excluded from the qualitative assessment [25].

**Table 1:** Studies reporting outcomes of emergency thoracotomy for trauma in Europe

References	Study period	Study design	*N*	Sex (M:F)	Age (years)	TRISS-score (Ps)	ISS (median, range)	MOI B:P	Survivors, *n* (%) B:P
Hudorovic, Croatia, Zagreb (2008) [21]	1995–2005	R	21	n.r.	n.r.	n.r.	n.r.	0:21	0:10 (47.6 %)
Søreide et al., Norway, Stavanger (2007) [19]	2001–2005	R	10	7:3	51 (range 21–77)	4 % (0–90 %)	35 (25–75)	7:3	0:0 (0 %)
Ferris et al., Scotland, Edinburgh (2008) [22]	2003–2005	R	6^a^	n.r.	n.r.	n.r.	n.r.	0:6	0:1 (16.7 %)
Pahle et al., Norway, Oslo (2010) [20]	2001–2007	R	109	75:34	30 (24–47)	6 % (0.1–22 %)	38 (26–50)	82:27	10:10 (18.3 %)
Kandler et al., Denmark, Copenhagen, (2012) [16]	2000–2009	R	44	40:4	32 years ± 14^b^	44 ± 32 %^b^	34 (IQR 25–48)	16:28	5:21 (59.1 %)
Van Waes et al., The Netherlands, Rotterdam (2012) [17]	2000–2011	R	56	48:8	32 (IQR 25–41)	n.r.	25 (16–34)	0:56	0:36 (64.3 %)
Johannesdottir et al., Iceland, Reykjavik (2013) [18]	2005–2010	R	9	9:0	36 (range 20–76)	85 % (1–96 %)	29 (16–54)	5:4	3:2 (55.6 %)
Lustenberger et al., Switzerland, Zurich (2012) [15]	1996–2008	R	121	92:29	38 (range 16–84)	n.r.	41 (16–70)	83:38	31:32 (52.1 %)

Data are reported as median with ranges unless otherwise specified

*N* number, *M:F* male:female, *MOI* mechanism of injury, *ISS* Injury Severity Score, *B:P* blunt:penetrating, *n.r.* denotes not reported, *n.a.* not applicable, *R* retrospective, *IQR* interquartile range

^a^Excluding 10 patients who underwent a procedure in the operating room due to lack of details

^b^Reported as mean with standard deviations (mean ± SD)

The 8 studies reported a total number of 183 penetrating and 193 blunt resuscitative emergency thoracotomies, varying from 9 cases in Reykjavik, Iceland to 121 cases from Zurich, Switzerland. Among the included studies (Table 1), four studies [15–17, 20] accounted for 88 % of the patients included. For studies reporting gender, the male:female ratio (271 males, 78 females) was 3.5:1 (Table 1). Mean age varied with more than two decades among studies, from lowest at 31 years to highest at 51 years.

Five studies reported the selected method of ET [15–18, 20]. Four studies from Denmark [16], Norway [19], the Netherlands [17] and Switzerland [15] included both emergency department and operating room thoracotomies and stated these were done for resuscitative purposes. In the Edinburg study [22], 6 patients had ERT in the ED and a further 10 in the OR. For the latter 10 procedures, no further descriptions were given and these 10 cases were thus excluded. Vital signs (systolic blood pressure, respiratory rate, pulse) were reported in 6 studies, and revised trauma score (RTS) in three studies from Iceland [18], Norway [19], and the Netherlands [17].

### MOI and LOMI

**Fig. 2:**
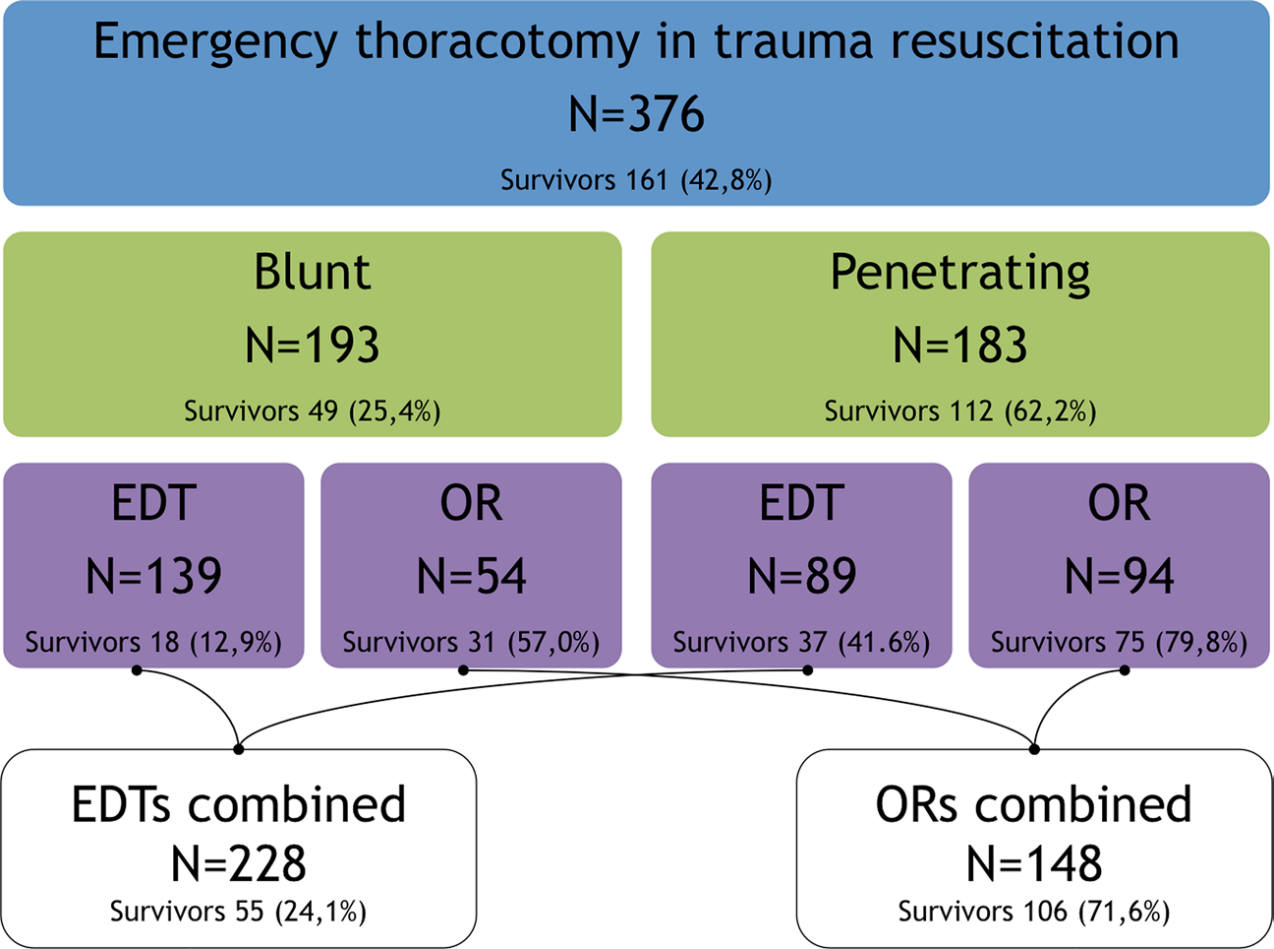
Breakdown of injury type, location and outcomes of the included patients in the identified studies. *EDT* Emergency department thoracotomies, *OR* operating room

MOI is reported in Table 1 and Fig. 2. Studies reporting LOMI, or related location of severe injuries are depicted in Table 2. Three studies reported specific injuries found during operation, without giving the LOMI [15–17].

**Table 2:** Reported LOMI for the included studies

References	*N*	Cerebral	Thoracic	Abdominal	Pelvic	Multiple
Croatia, Zagreb (2008) [21]	21	n.r.	21 (cardiac)	n.r.	n.r.	(unknown)^a^
Norway, Stavanger (2007) [19]	10	1	4	1	0	4
Scotland, Edinburgh (2008) [22]	6	–	6	–	–	–
Norway, Oslo (2010) [20]	109	n.r.	n.r.	n.r.	n.r.	n.r.
Denmark, Copenhagen (2012) [16]	44	n.r.	n.r.	n.r.	n.r.	n.r.
Netherlands, Rotterdam (2012) [17]	56	–	56	(10)		10
Iceland, Reykjavik (2013) [18]	9	–	6	–	–	3
Switzerland, Zurich (2012) [15]	121^b^	50	118	61	n.r.	n.r.

^a^Associated extra-thoracic injuries were noted as a prognostically poor variable

^b^Reported all injuries with AIS ≥3 for each body region

### Indication for ERT

All studies described an indication for ERT, but with variable degree of information and whether or not this was done according to a pre-stated institutional protocol. In the Croatian cohort, all were done for thoracic or suspected cardiac injuries, but with no other description available. The indication for each ERT that was performed was described in the other studies. The studies from Scotland and the Netherlands both included only penetrating injuries, of which the majority to the chest and with some form of physiological compromise. For blunt trauma, motor vehicle collisions and falls predominated, while stab wounds were more frequent than gunshots wounds for penetrating trauma (data not shown). Johannesdottir, Ferris and Soreide describe the indication as either suspected tamponade, cardiac arrest; and, witnessed loss of SOL or suspected exsanguination [18, 19, 22].

Pahle [20] stated the indications as either “unresponsive patient with penetrating injury who has shown SOL during transport or at the scene”; “exsanguinated patients without immediate response to fluid resuscitation”; and “obviously large abdominal bleeding and decreasing blood pressure with no response to fluid resuscitation before laparotomy”, but with no further specification to the distribution in specific patients.

Kandler et al [16] describe ‘penetrating trauma with pulseless electric activity (PEA) within the last 5 min, unstable patients with ongoing intrathoracic bleeding, and as means of clamping the descending thoracic aorta as a step in the initial resuscitation’, as indications for ERT [16].

Van Waes et al. [17] give the most detailed indication for ERT: (1) Loss of SOL on arrival ED, but present at scene; (2) failure to respond to resuscitation with SBP <60 mmHg, or pericardial tamponade and SBP <60 mmHg; (3) Hemothorax on chest X-ray and initial output >1500 mL or ongoing output of >200 mL/h for 2–4 h after insertion of the tube; (4) Hemothorax on chest X-ray, with <1500 mL, but CTA findings prompting surgical intervention; (5) Massive air embolism.

Lustenberger et al. [15] used the indication ‘non-recordable blood pressure on ED admission, loss of SOL in the ED or immediately before hospital arrival and exsanguination from trauma without immediate response to fluid resuscitation’.

### Prehospital factors and transport time

Time variables were reported in different ways and for different time intervals across the studies. Hudorovic [21] noted a difference in median prehospital transport time in survivors (median 150 min, range 15–180 min) compared to non-survivors (median 220 min, range 30–220 min). Pahle et al. [20] did not find a significant difference in survivors and non-survivors, when assessing time from injury to arrival in ED; non-survivors arrived within a median of 40 min (IQR 18–84 min) compared to survivors who arrived in ED a median of 45 min (IQR 25–95 min) after injury (*P* = 0.477). The same non-significant findings occurred for those with penetrating injuries in the same study, with transport time to ED being almost reduced by half to 20 and 27 min for non-survivors and survivors, respectively [20]. This time interval matched with the penetrating cohort from the Dutch group having a prehospital transport time at median 24 min (IQR 15–32) overall, but with significant difference in transport time between patients who went on to have ERT in the emergency department compared to the operating room (median 13 min (IQR 2–23) compared to 33 min (IQR 18–35; *P* = 0.006). Of note, the median time until actual ET was performed was 68 min (IQR 42–128) for the whole group; with less time passing to ERT in the ED (median 25 min (IQR 15–107)) compared to the operating room ERTs (median 79 (IQR 52–155) for a *P* = 0.037 [17].

In the Swizz study [15], the median time from ED admission to the start of ERT in the ED was 5 (range 2–17) min, and the median time from ED admission to the start of ERT in the OR was 25 (15–49) min.

### Sign of life

Six out of 8 studies reported SOL [15–20] (Table 1). According to one publication from Norway [19], 7 of 10 (70 %) patients had SOL at scene, but only 4 out of 10 patients (40 %) had SOL in the ED. In the second study from Norway, 86 of 109 (79 %) had SOL at injury site, but SOL in the ED is not given [20]. In Danish cohort, 19 of 21 (90.5 %) had SOL in ED [16]. In the Dutch publication 55/56 (98 %) had prehospital SOL, and 50/56 (89 %) showed SOL in hospital [17]. In the Iceland study, 6 of 9 (66.7 %) had SOL, but it is not described in detail if this was in ED. However, the 3 patients without SOL all died [18]. In the Swizz study [15], SOL is reported “at scene” 46/49 EDT (94 %), “en route” 40/49 (82 %) and “on admission” 33/49 (67 %) for those procedures that were performed in as EDT, but not mentioned for procedures performed in the OR. The Croatian report states “absence of SOL at the hospital was a herald of mortality”, but mentions no distribution of SOL from their data [26]. The Scottish study does not comment on SOL [22].

### Location of ERT

Regarding the procedures performed in the OR, the Danish report [16] stated that these were included if considered part of the immediate resuscitative process, but did not give any criteria for performing the ERT in the ED or OR. Similarly, the Swizz study stated that EDT was performed for patients in extremis, while those with pending shock or who were deemed to tolerate transport would be performed in the OR as the immediate resuscitative procedure [15]. The same indications for ED and OR were noted in one Norwegian study [19]. The Dutch group [17] performed the procedure in ED if systolic blood pressure (SBP) was <60 mmHg (50 % of cases), presence of cardiac tamponade or, loss of SOL in the ED (41 %). Procedures taken to the OR had SBP >60 mmHg (but <100 mgHg), initial chest output >1500 mL blood, or ongoing chest tube output >200 mL/h (together 50 % of cases in OR), pericardial tamponade with SBP >60 mgHg (27 %). Most procedures were performed as an anterolateral thoracotomy, followed by sternotomy (Table 3).

**Table 3:** Procedure type performed for ERT

References	*N*	Anterolateral	Median sternotomy	Combined	Clam shell	Posterolateral
Norway, Stavanger (2007) [19]	10	10 (100 %)	n.r.	n.r.	n.r.	n.r.
Norway, Oslo (2010) [20]	109	74 (68 %)	10 (9 %)	25 (23 %)	–	–
Denmark, Copenhagen (2012) [16]	44^a^	33 (75 %)	5 (11 %)	4 (9 %)	–	–
The Netherlands, Rotterdam (2012) [17]	56^b^	30 (54 %)	22 (39 %)	–	4 (7 %)	8 (14 %)
Iceland, Reykjavik (2013) [18]	9	5 (56 %)	2 (22 %)	2 (22 %)	–	–
Switzerland, Zurich (2012) [15]	121	81 (67 %)	28 (23 %)	8 (7 %)	4 (3 %)	–

*n.r.* not reported

^a^2 missing data for surgical approach

^b^The authors state that total 64 incisions were performed

### Survival

The collectively reported overall survival for all 376 ERTs is presented in Fig. 2, with breakdown in MOI and location of where performed (ED or OR) with survival rates. For blunt trauma survival was 25.4 % and for penetrating injuries 62.2 % (Fig. 2). Three out of the 5 articles reporting on blunt trauma ERT had survival rates above 10 % [16, 18, 20], ranging from 12.2 % [20] to 60.0 % [18]. When strictly including only those ERTs designated as done in the ED and for blunt injury (*n* = 139), the survival was 12.9 % (*n* = 18).

As noted in Table 1, only a few studies reported probability of survival and with considerable spread in the estimates.

### Neurological outcome in survivors

Neurological outcome was reported in 5 of 8 studies [15, 17, 18, 20, 26], most with favorable neurological long-term outcome in survivors, even in blunt trauma survivors. This was reported in both low volume and high volume studies. Among the 8 publications representing a total of 161 survivors after ERT none of the investigators reported neurological outcome using the Glasgow Outcome Scale (GOS) [14] or similar objective measures. Rather, a qualitative designation as “poor” or “good” neurological outcome or ‘neurologically intact’ or ‘without neurological impairment’ was used in most studies. For 34 survivors, no statement on neurological status or outcome was made. Thus, outcome was available in 127 (78.9 %) of survivors, of which 86.6 % had a satisfactory or good neurological recovery. In 17 survivors, the outcome was designated as persistent neurological impairment or inability to live an independent life. One study [26] reported neurological impairment in all survivors (*n* = 10) with none living independent lives after survival from ERT.

## Discussion

This systematic review found 8 studies on ERT for trauma in European civilian trauma populations. Collectively, these accumulated survival rates are higher than previously reported in the literature—even for blunt trauma performed in the emergency department survival was 12.9 %. The findings are at variance with the perceived standards and outcome reported by major reports, reviews and guidelines previously [2, 5, 27, 28]. These studies report a much higher survival rate than previously reported and may indeed point to a less futile outcome for blunt trauma victims with pending cardiac arrest, witnessed loss of SOL and even who undergo cardiopulmonary resuscitation than previously argued for in most studies [2–4, 27, 28].

The role of ERT continues to stir considerable debate [5–7, 27–31]. Proponents have even suggested taking the procedure to the pre-hospital field for selected patients in extremis [9, 29]. While all agree that blunt mechanism is in principle associated with a disfavorable prognosis, there are some reports on favorable outcome in a few selected patients, thus arguing for a not completely nihilistic view even in those with blunt trauma [28, 31]. However, it is clear that more knowledge is needed in this area. Further, one may envision the indication of resuscitative emergency thoracotomy for trauma to change with the increasing availability of resuscitative endovascular balloon occlusion of the aorta (REBOA) technique [32]. However, this is currently performed only in some specialized centers.

Moore et al. [6] reported duration of prehospital CPR as a reliable means to establish futility for ERT. Notably, in a large German registry study of over 10,000 trauma patients, a limited number of 757 patients had documented prehospital cardiopulmonary resuscitation with on-scene or en route performed closed chest compression due to traumatic arrest [25]. The rate of emergency thoracotomy in this cohort was 10.2 % (*n* = 77) for a reported survival rate of 13.0 % (95 % CI 5.5–20.5), including both penetrating and blunt trauma [25]. From a registry perspective, this falls within the cumulatively reported range of 12.6 % survival for blunt injury ERT in this study (for those performed in ED alone).

The overall frequency by which lifesaving or hemostatic emergency surgery procedures are performed among injured patients is hard to tell from the worldwide literature. However, another updated German nationwide study of almost 13,000 trauma patients found immediate lifesaving procedures (performed during resuscitation) in 5.5 % of patients [33]. Among the 713 patients that had interrupted resuscitation for an emergent procedure, this most frequently was done as a laparotomy (51 %), followed by craniotomy in 20 %, and only 10 % had thoracotomy (and 9.3 % had pelvic surgery) [33]. In the German report, 70 procedures were emergency thoracotomies and lethality was 50 %. Notably, of the 70 ETs, 16 were for cardiac injury, of which only 5 survived (32 %) [33]. In a study from Northern Norway (*n* = 142) [23], <3 % of all patients required hemostatic surgery on admission, and only 5 patients had an emergency thoracotomy, with no survivors. In another study from Dublin [24], 5 % of penetrating trauma victims had a thoracotomy, although no further data was provided on indication nor on outcome in this report. However, these reports indicate that emergency salvage procedures are not commonly performed in European trauma populations [23, 24, 33].

The number of relevant articles from European trauma hospitals on ERT is limited, as demonstrated by this systematic review. Our search only found 8 articles that fulfilled the criteria. Consequently, we cannot rule out a publication bias that may skew the results. Four studies accumulated 88 % of the entire cohort and may bias the results accordingly. However, the studies are from diverse geographic regions, with variable volume and difference in trauma systems. Also, as mentioned above, they match with experience reported from nationwide registry, such as the German Trauma registry database.

The report from Iceland on 60 % survival for blunt trauma included few (*n* = 5) patients. In contrast, Pahle and Lustenberger, respectively, had 82 blunt and 39 blunt cases, with 12.2 and 7.7 % blunt trauma survivors after ERT, respectively. This could point to a higher rate of survival in mature trauma system. Also, it may reflect an aggressive approach to emergency thoracotomy where one may entertain the possibility that some patients would have survived without being exposed to the ERT procedure. Accordingly, and as a flipside to that coin, hospitals with a more restrictive approach will have fewer survivors after ERT, in particular for blunt trauma. These nuances cannot be discerned from the available data, but certainly warrants future validation in other series before any firm conclusions can be made.

There are some limitations to this study that warrants mentioning. One is the selected reports available from a few centers of variable size and geography. While some reports stem from larger European urban areas such as Oslo, Copenhagen, Zurich and Rotterdam, there is a paucity of similar reports from larger, metropolitan regions in Europe where a large number of trauma patients are treated. As mentioned, a publication bias may thus exist. For example, little is known about ERT performed in major civilian trauma patients in the UK (London, Birmingham), Germany (Berlin, Munich), the Netherlands (Amsterdam, Utrecht), Spain (Barcelona, Madrid), Italy (Rome, Milan) or France (Paris, Marseilles), to mention but a few. It is thus not known if the findings from the selected studies are representative of Scandinavia, the British Isles or mainland Europe, as such. Further, there is considerable variation in the reports, with inconsistent data reporting among the studies. Of notice, all the reported studies were retrospective in design. Thus, use of other definitions, variable data records and non-recorded or missing data may introduce bias in the reported studies.

Despite these limitations, this is the first study to collectively review the experience and outcome of ERT in European civilian trauma patients in the 21st century. It calls into questions the somewhat nihilistic view expressed toward resuscitative emergency thoracotomy for blunt trauma. Further studies are warranted to explore the generalizability of the findings, in particular for blunt trauma.

## Conclusions

In this collective, systematic review of European studies, half of procedures were for blunt trauma, with survivors after ERT in the ED collectively reported at 12.9 and 41.6 % for blunt and for penetrating injuries, respectively. There was no firm indication that neurological outcome were dismal, but further studies need to confirm these results, as considerable variation to previous collective reports exists. A protocolized, multicenter, prospective observational study should be launched to address these questions and arrive at better answers for correct indications and related outcomes of ERT in severely injured trauma patients.
